# Household solid fuel use and stroke incidence: Evidence from a national cohort study

**DOI:** 10.3389/fpubh.2022.1018023

**Published:** 2022-10-20

**Authors:** Hehua Zhang, Yang Xia, Han Su, Qing Chang, Yuhong Zhao

**Affiliations:** ^1^Clinical Research Center, Shengjing Hospital of China Medical University, Shenyang, China; ^2^Department of Clinical Epidemiology, Clinical Research Center, Shengjing Hospital of China Medical University, Shenyang, China; ^3^Department of Health Management, Shengjing Hospital of China Medical University, Shenyang, China; ^4^Liaoning Key Laboratory of Precision Medical Research on Major Chronic Disease, Shengjing Hospital of China Medical University, Shenyang, China

**Keywords:** household air pollution, solid fuel use, stroke, cohort study, public health

## Abstract

Stroke is one of the leading causes of global mortality and disability. No specific study has focused on the association between household solid fuel use for different purposes and incident stroke. Therefore, we explored the associations between household solid fuel use purposes and switches and incident stroke based on a national prospective cohort study. There were 12,485 participants included in this study after exclusions. The incidence density of stroke was 8.29 for every 1,000 person-years. Household solid fuel use simultaneously for heating and cooking had the largest hazard effect on stroke occurrence [hazard ratio (HR), 1.35; 95% confidence interval (CI), 1.07, 1.70] with a significant linear trend (*P* < 0.01). Solid fuel use for cooking was significantly associated with increased risk of stroke occurrence (HR, 1.27; 95% CI, 1.06, 1.51). Persistent clean fuel use for both heating and cooking associated with a lower risk of stroke occurrence (HR, 0.79; 95% CI: 0.64, 0.99), and switching from solid fuel to clean-fuel use for cooking associated with a lower risk of stroke occurrence (HR, 0.89; 95% CI, 0.73, 1.09) compared with persistent solid fuel use. Effective measures to improve the household cooking environment may be necessary to prevent incident stroke.

## Introduction

Stroke is a sudden, focal neurologic deficit that can be divided into ischemic stroke and intracerebral hemorrhage from vascular origin and confined to brain or eye (1). According to the study of 2019 Global Burden of Disease (2), stroke is the second leading cause of global mortality and disability, and China has one of the highest age-standardized rates of mortality and number of disability-adjusted life years due to stroke, ranging from 16.8 to 20.7%. Risk factors for stroke include, but are not limited to elderly population, female, and chronic diseases, such as hypertension, diabetes, dyslipidemia, and heart attack, among others (3). Discovering new risks is of great importance for preventing the occurrence of new stroke events.

Air pollution from ambient and household, currently poses a considerable environmental burden on global health, especially in terms of respiratory and cardiovascular diseases (4). Ambient air pollution exposure has been associated with an increased risk of stroke in several large cohort studies (5). The components of ambient and household air pollution are different (6). Many stroke patients come from rural villages in China (7), and they engage in heavy household solid fuel use (such as using coal or wood for cooking and heating) as opposed to being exposed to ambient air pollution. Solid fuel use is the chief source of household air pollution in rural China (8), thus, contributing to heavy household particulate-matter pollution, as seen in previous studies (9). Household solid fuel use is associated with an increased risk of several chronic diseases, such as hypertension, arthritis, depression, and diabetes (10–12). Thus, it is important to explore the association between household solid fuel use and stroke occurrence, especially in rural China. To the best of our knowledge, only one longitudinal study has reported an association between solid fuel for heating and an increased risk of stroke in non-smoking Chinese individuals (13). One recent cohort study reported solid fuel use for cooking increased incident non-fetal cardiovascular disease (14), however, this study mix stroke with heart disease. Two cohort studies have reported an association between household coal use and an increased risk of stroke mortality, which were limited to a small sample (15) and the latter exclusively focused on women (16). Switching behaviors on fuel types could receive health benefits, which has been proposed by previous studies (17, 18). No specific studies have focused on the relationship between household solid fuel use for different purposes and the incidence of stroke events.

Therefore, we aimed to explore the relationships between different household solid fuel use purposes as well as switches of fuel use types and stroke occurrence based on a national longitudinal cohort study.

## Materials and methods

### Participants

**Figure 1:**
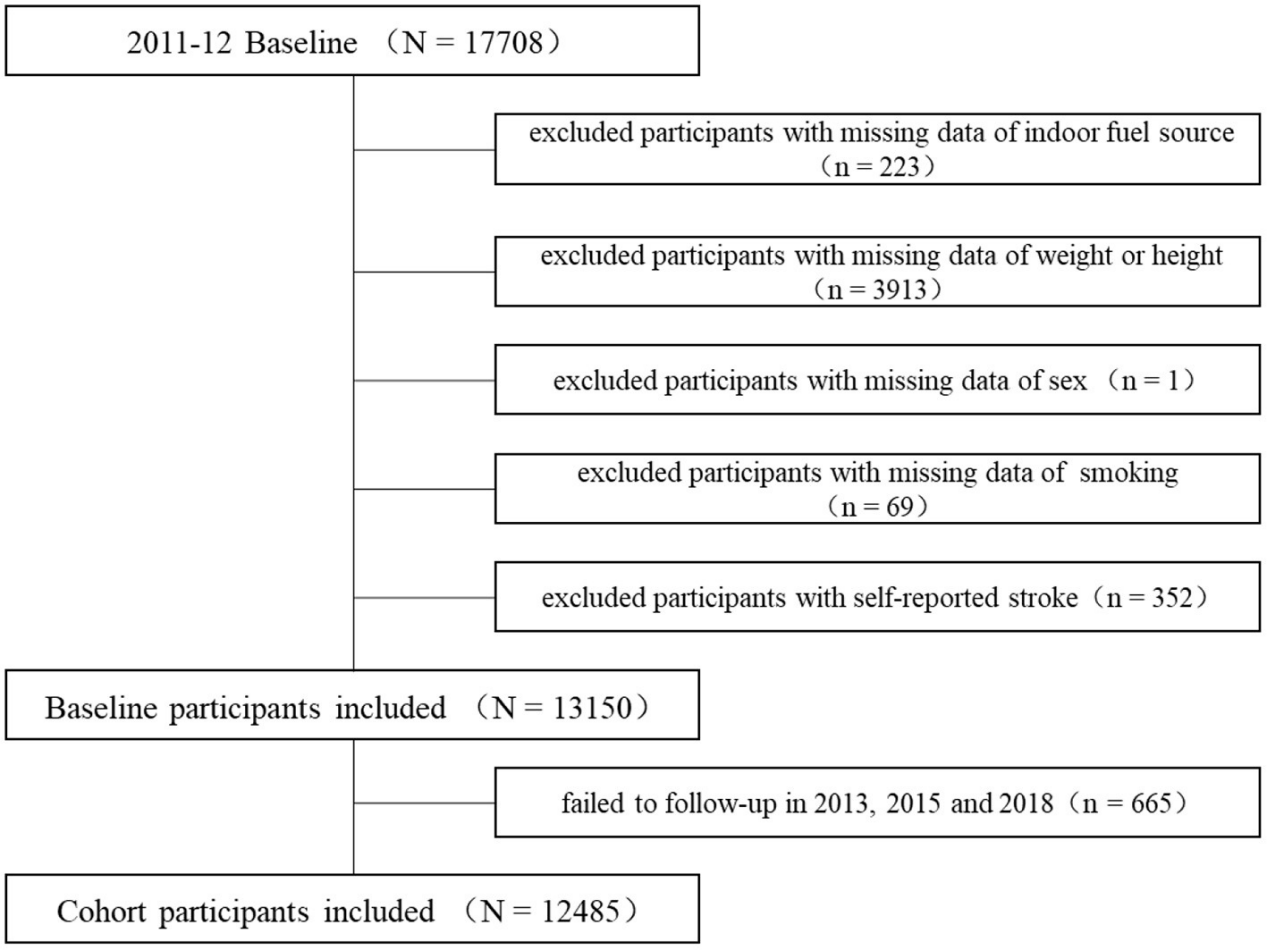
Participant-selection process.

The participants were from the China Health and Retirement Longitudinal Study (CHALRS), the details of which have been described elsewhere (19). The baseline investigation of 17,708 participants from 28 provinces was conducted in 2011–2012, and follow-up investigations were conducted biennially in 2013, 2015, and 2018. We excluded participants as follows: with missing data on household fuel use (*n* = 223), weight or height (*n* = 3,913), sex (*n* = 1), and smoking behavior (*n* = 69) as well as those with self-reported incident stroke (*n* = 352). We further excluded 665 participants with no clear outcome of stroke events in any of the three follow-up investigations. The detailed participant selection process is presented in Figure 1. This study was approved by the Ethics Committee of Peking University, and each participant signed an informed consent form.

### Stroke definition

Face-to-face interviews were conducted between trained investigators and each participant (or his/her spouse). Participants were requested to respond to the question, “Have you ever been diagnosed with stroke by a doctor?” in baseline or “Have you been diagnosed with stroke by a doctor since your last interview?” in follow-ups, Response options included “Yes,” “No,” and “Not clear.” Participants with a precise “No” answer in baseline and a precise “Yes” answer during the follow-up investigation period were classified as having incident stroke outcomes. Participants with a precise “No” answer during the baseline and follow-up investigation period were classified as having no incident stroke outcome. Participants without a precise “Yes” or “No” answer during the baseline or follow-up investigations were excluded from this study.

### Household fuel use

Data on household fuel use were collected during the baseline questionnaire survey. Participants were requested to indicate their main heating and cooking energy sources. The use of energy sources such as “coal” and “crop residue/wood” was considered solid fuel use for heating or cooking, whereas that of heating energy sources such as “solar,” “natural gas,” “electricity,” or “liquefied petroleum gas” was considered clean fuel use for cooking or heating. Data on the alteration of household fuel use for heating and cooking compared with that on baseline household fuel use were collected from follow-up investigations in 2013–2018, and they were categorized into four classes as follows: “solid at both baseline and follow-up,” “clean at follow-up and solid at baseline,” “solid at follow-up and clean at baseline” and “clean at both baseline and follow-up.”

### Other variables

Variables that were potential confounding factors for the association between household solid fuel use and incident stroke were included in this study. Data on variables, including age, sex, family income, education level, marital status, smoking and drinking behavior, home location, chronic diseases, retirement status, weight, and height, were collected during the baseline face-to-face questionnaire survey. Education level was categorized into three types as follows: low (illiterate, did not finish primary school or finished elementary school), middle (did not finish or finished middle school, high school, or vocational school), and high (did not finish or finished college or above). Body mass index (BMI) at baseline was defined as the participant's weight in kilograms (kg) divided by the square of his/her height in meters (m^2^). Home locations were categorized into urban or rural villages. Marital status was classified as married (living together) or single (separated, divorced, or widowed). Chronic diseases refer to self-reported chronic diseases diagnosed by a doctor, including hypertension, diabetes or high blood sugar, dyslipidemia, heart attack, liver disease, emotional problems, kidney disease, arthritis, chronic lung diseases, asthma, and memory-related diseases. An average outdoor exposure concentration of PM_2.5_ (particulate matter with a diameter <2.5 μm) at city level between 2011 and 2018 was calculated as a confounding factor in this study, and the original PM_2.5_ data, obtained from the National Aeronautics and Space Administration, has been described in detail elsewhere (20).

### Statistical analysis

The characteristics of the included participants in baseline were described according to their stroke status at follow-up. Chi-square tests or variance analysis were used to examine the differences between baseline characteristics according to the distribution of categorized variables and numerical variables, respectively. Counts (percentages) and least-square means [95% confidence intervals (CIs)] were used to describe categorical and continuous variables, respectively. We used Cox proportional hazards regressions to explore the association between household solid fuel use and stroke occurrence. Four models were used. Crude model did not adjust any variables. Model 1 was adjusted for age, BMI and sex; Model 2 was further adjusted for education level, family income, marital status, home location (rural or urban area), number of chronic diseases, smoking and drinking behaviors, and retirement status based on Model 1; Model 3 was further adjusted for outdoor air-pollution exposure of PM_2.5_ based on Model 2. The linear trends for the associations between types of household solid fuel use and stroke occurrence were explored. *P*-values for interaction were calculated by including multiplicative terms for household energy sources and age [young elders (age ≤ 60 years) and old elders (age > 60 years)], household energy sources and sex, or household energy sources and smoking status (“never-smoker” or “smoker” [including “current” and “ever-smoker”]) in the Cox regression analysis. Subgroup analyses according to age, smoking status, or sex, were also conducted. For sensitivity analysis, associations between alterations of household fuel use for cooking during follow-ups and stroke occurrence were explored. The final models were adjusted for age, sex, BMI, education level, smoking and drinking behaviors, family income, marital status, retirement status, home location (rural or urban area), number of chronic diseases, and outdoor exposure to PM_2.5_. Hazard ratios (HRs) and 95% CIs are presented. All statistical analyses were performed using SAS (version 9.4; SAS Institute Inc., Cary, NC, USA).

## Results

### Baseline characteristics

Table 1 presents the characteristics of the participants in baseline according to stroke status at follow-up. A total of 12,485 participants were included in this cohort study. The incidence density of stroke was 8.29 for every 1,000 person-years. New stroke concurrences tended to be occurred in participants with older age (*P* < 0.0001), higher BMI (*P* < 0.0001), single (*P* < 0.01), lived in an urban community (*P* < 0.01), lower family income (*P* < 0.0001), retirement status (*P* < 0.0001), smoking behavior (*P* < 0.001), and more comorbid chronic diseases (*P* < 0.001).

**Table 1:** Baseline characteristics of participants.

**Characteristics**	**Stroke status**		***P-*value^a^**
	**Yes (*n* = 662)**	**No (*n* = 11,823)**	
Age, years	60.56 (59.83, 61.3)^b^	58.27 (58.1, 58.45)	<0.0001
BMI,	24.48 (24.18, 24.78)	23.43 (23.36, 23.5)	<0.0001
Sex, *n* (%)			0.67
Female	311 (46.98)^c^	5,455 (46.14)	
Male	351 (53.02)	6,368 (53.86)	
Marriage status, *n* (%)			0.11
Married	568 (85.80)	10,390 (87.88)	
Single	94 (14.20)	1,433 (12.12)	
Location, *n* (%)			<0.05
Urban community	261 (39.43)	4,212 (35.63)	
Rural village	401 (60.57)	7,611 (64.37)	
Family income, *n* (%)^d^			<0.0001
Low	599 (90.48)	9,953 (84.18)	
High	63 (9.52)	1,870 (15.82)	
Retirement, *n* (%)			<0.0001
No	451 (68.13)	8,894 (75.23)	
Yes	211 (31.87)	2,929 (24.77)	
Education level, *n* (%)			
Low	188 (28.40)	3,350 (28.33)	0.97
Middle	285 (43.05)	4,782 (40.45)	0.18
High	189 (28.55)	3,691 (31.22)	0.15
Smoking			
Never	389 (58.76)	7,272 (61.51)	0.16
Ever	78 (11.78)	949 (8.03)	<0.001
Current	195 (29.46)	3,602 (30.47)	0.59
Drinking			
≥Once a month	168 (25.38)	2,934 (24.82)	0.74
<Once a month	45 (6.80)	928 (7.85)	0.33
Never	449 (67.82)	7,961 (67.33)	0.72
Comorbidities			
No	128 (19.34)	3,966 (33.54)	<0.0001
One disease	187 (28.25)	3,677 (31.10)	0.12
≥ Two diseases	347 (52.42)	4,180 (35.35)	<0.0001
Outdoor PM_2.5_ concentration (μg/m^3^)^e^	38.13 (36.91, 39.36)	37.6 (37.31, 37.88)	0.40

a Variance analysis or Chi-square tests.

b Least square mean (95% CI) (all such values).

c Count (percentage) (all such values).

d A family income lower than the median (4,114 RMB) was classified as low income.

e Average concentration in 2011–2018 at the city level.

### Association between household solid fuel use and stroke occurrence

There were 7,845 and 6,068 participants using solid fuel for heating and cooking, respectively. As shown in Table 2, participants were categorized according to solid fuel use types. Compared with clean-fuel use for both heating and cooking, solid fuel use for both heating and cooking had the largest hazard effect on stroke occurrence (HR, 1.35; 95% CI, 1.07, 1.70), with a linear trend (*P* < 0.01). As shown in Table 3, solid fuel use for heating and cooking was associated with a higher risk of stroke than clean fuel use. Solid fuel use for cooking was associated with an increased risk of stroke occurrence (HR, 1.27; 95% CI, 1.06, 1.51).

**Table 2:** Associations between household solid fuel use and stroke occurrence.

**Household solid fuel use type^e^**	**HR (95% CI)** ^**d**^			
	**Crude model**	**Model 1^a^**	**Model 2^b^**	**Model 3^c^**
Both clean (*n* = 2,543)	Ref	Ref	Ref	Ref
Either solid (*n* = 3,351)	0.97 (0.76, 1.22)	1.01 (0.79, 1.27)	1.07 (0.84, 1.36)	1.07 (0.84, 1.36)
Both solid (*n* = 6,591)	1.16 (0.95, 1.42)	1.18 (0.96, 1.45)	1.34 (1.06, 1.69)	1.35 (1.07, 1.70)
*P* for trend	0.07	0.06	<0.01	<0.01

a Adjusted for age, sex, and BMI.

b Further adjusted for family income, education level, smoking and drinking behaviors, marital status, home location (rural or urban area), number of chronic diseases, and retirement status, based on Model 1.

c Further adjusted for outdoor air-pollution exposure of PM_2.5_, based on Model 2.

d Cox proportional hazards regression models.

e Clean: clean fuel used for both cooking and heating, either solid or clean: one clean fuel and one solid fuel used for cooking and heating, solid fuel used for both cooking and heating.

**Table 3:** Associations between purposes of solid fuel use and stroke occurrence.

**Household solid fuel use**	**HR (95% CI)** ^**d**^			
	**Crude model**	**Model 1^a^**	**Model 2^b^**	**Model 3^c^**
For heating (*n* = 9,326)	1.11 (0.93, 1.33)	1.13 (0.94, 1.36)	1.21 (0.99, 1.47)	1.13 (0.92, 1.39)
For cooking (*n* = 7,207)	1.17 (1, 1.36)	1.16 (0.99, 1.36)	1.27 (1.07, 1.51)	1.27 (1.06, 1.51)

a Adjusted for age, sex, and BMI.

b Further adjusted for family income, education level, smoking and drinking behaviors, marital status, home location (rural or urban area), number of chronic diseases, and retirement status, based on Model 1.

c Further adjusted for other household solid fuel uses (for cooking or heating) and outdoor air-pollution exposure to PM_2.5_, based on Model 2.

d Cox proportional hazards regression models.

### Alteration of household solid fuel use for cooking and stroke occurrence

In terms of the limited data regarding heating energy sources during follow-up in 2015 and 2018, we only analyzed the alterations of household solid fuel use for cooking and stroke occurrence. A total of 2,781 participants used solid fuel for cooking at baseline and switched to clean-fuel use during the 7-year follow-up period. A total of 2,011 participants were excluded from the analysis because of missing data on cooking energy sources during the follow-up period. A total of 512 participants using clean fuel for cooking switched to solid fuel use during the follow-up period, and they were excluded from the analysis owing to the limited numbers and illogical alteration of cooking fuel use. As shown in Table 4, there was a linear trend (*P* = 0.04) indicating that clean-fuel use for cooking had a protective effect on stroke occurrence. Compared with those who persistently used solid fuel for cooking, both at baseline and during the follow-up period, participants who persistently used clean solid fuel had a lower risk of stroke occurrence (HR, 0.79; 95% CI, 0.64, 0.99), and switching from solid fuel use for cooking to clean-fuel use resulted in a lower risk of stroke occurrence (HR, 0.89; 95% CI, 0.73, 1.09).

**Table 4:** Associations between alteration of household solid fuel use for cooking and stroke.

**Alteration of cooking fuel use**	**Crude model**	**Model 1^a^**	**Model 2^b^**	**Model 3^c^**
Solid in both baseline and follow-up (*n* = 3,858)	Ref	Ref	Ref	Ref
Solid in baseline and clean in follow-up (*n* = 2,781)	0.92 (0.76, 1.13)^d^	0.94 (0.77, 1.14)	0.89 (0.73, 1.09)	0.89 (0.73, 1.09)
Clean in both baseline and follow-up (*n* = 3,323)	0.88 (0.73, 1.06)	0.89 (0.74, 1.08)	0.78 (0.63, 0.96)	0.79 (0.64, 0.99)
*P* for trend	0.17	0.23	0.03	0.04

a Adjusted for age, sex, and BMI.

b Further adjusted for family income, education level, smoking and drinking behaviors, marital status, home location (rural or urban area), number of chronic diseases, and retirement status, based on Model 1.

c Further adjusted for outdoor air-pollution exposure of PM_2.5_, based on Model 2.

d Hazard ratio and 95% CI calculated from Cox proportional hazards regression models.

### Modifying effect analyses according to of age, sex, and smoking behavior

**Figure 2:**
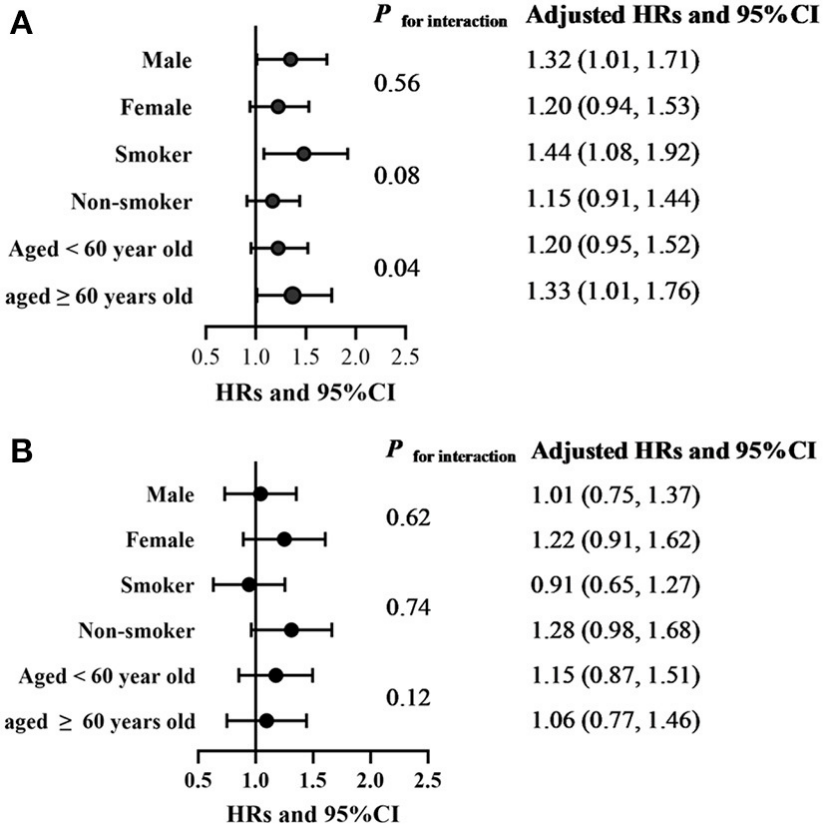
Subgroup analysis of the associations between solid fuel use and stroke according to age, sex, and smoking status. Cox proportional hazards regression adjusted for age, sex, BMI, family income, education level, smoking and drinking behaviors, marital status, home location (rural or urban area), number of chronic diseases, retirement status, other household solid fuel uses and outdoor air-pollution exposure to PM_2.5_. **(A)** Analysis of the associations between solid fuel use for cooking and stroke. **(B)** Analysis of the associations between solid fuel use for heating and stroke.

Subgroup analysis of the associations between solid fuel use and stroke according to age, sex, and smoking status was presented in Figure 2. Modifying effect of age on the association between household solid fuel use for cooking and incidence of stroke was found (*P* for interaction = 0.04). Sub-group analysis indicated that incident stroke was more affected by household solid fuel use for cooking in participants aged over 60 years old (HR, 1.33; 95%CI, 1.01, 1.76), in smokers (HR, 1.44; 95% CI, 1.08, 1.92) and in male participants (HR, 1.32; 95% CI, 1.01, 1.71). There was no evident difference in the associations between household solid fuel use and stroke incidence among different sub- groups.

## Discussion

This was the most comprehensive cohort study to explore the associations between household solid fuel use for different purposes and stroke incidence in general Chinese adults up to now. The findings of this study indicate that household solid fuel use for cooking, but not heating, is associated with higher risk of stroke. Switching from solid fuel use to clean-fuel use for cooking is associated with a lower stroke incidence. Sub-group analyses revealed associations between solid fuel use for cooking and stroke incidence tended to be in male and smoking participants.

Previous studies have predominantly focused on the association between outdoor air pollution exposure to certain air pollutants and stroke, and their findings indicate that exposure to outdoor air pollutants, such as PM_2.5_, sulfur dioxide (SO_2_), and nitrogen dioxide (NO_2_), is associated with an increased risk of stroke. Studies on the effects of household solid fuel use on stroke incidence are limited. A cohort study from the China Hypertension Survey involving 13,528 non-smoking participants (among whom were 273 participants with stroke) found solid fuel use for heating to be independently associated with an increased risk of fatal or non-fatal stroke incidence (HR, 1.44; 95% CI,1.00, 2.08) (16), no results of solid fuel use for cooking and stroke were reported; Furthermore, the main results were different from the findings in this study, subgroup analysis were similar and found non-smoking participants were more affected by household solid fuel use. The high incidence of stroke in this study might have also been owing to the long follow-up period (mean, 6.39 years). A cross-sectional study involving 1,078 households among Thailand villagers reported that solid fuel use for cooking was associated with an increased risk of stroke incidence [using wood: odds ratio (OR), 7.64; 95% CI, 1.18, 49.61; using charcoal: OR, 3.17; 95% CI, 1.04, 9.71] (21). A small-sized cohort study conducted in 1998 in Shanghai of China (17) reported that household coal-fume exposure was associated with an increased risk of stroke incidence (Relative risk, 9.72; 95% CI, 1.88, 50.24). Except for the great significance of these studies, there were large confidence-intervals in the study probably due to small sample size. A study from the Xuanwei cohort in China involving 20,179 non-smoking women (18) reported that an increased risk of stroke mortality was observed among lifetime users of smokeless coal (across lifetime, >196 ton-years, HR, 1.90, 95% CI, 1.10, 3.30). Since smokeless coal was commonly used as an household heating fuel, the results of the Xuanwei cohort were similar to those of this study's subgroup analysis, in which the stroke risk in female participants was more affected by solid fuel use for heating than in male participants.

The mechanism underlying the relationship between household solid fuel use and stroke remains unknown. Higher inflammation and oxidative stress caused by household solid fuel use may be the major biological mechanisms, which promote aging and cerebral vasculature and increase the risk of atherosclerotic ischemic stroke (22). Considering that stroke is the major adverse outcome of hypertension, the biological mechanism underlying the relationship between solid fuel use and stroke may be similar to that between household air pollution and hypertension. The elevation of stress-hormone levels caused by particulate matter from solid fuel use may be a biological mechanism (23), and a previous study reported that greater stress was associated with an elevated risk of incident stroke in patients with hypertension (24). Airborne nanoscale particles from household solid fuel use may also cause an abnormal expression of coagulation factors, resulting in coagulation cascades and incidents of coagulation-related cardiovascular diseases, including stroke (25), which is another potential mechanism underlying the relationship between household solid fuel use and stroke incidence.

According to previous studies, the effect of household air pollution on health outcomes could be modified by confounding factors, such as sex and smoking status; thus, we conducted interaction and subgroup analyses between solid fuel use and age (or sex or smoking status). We did find interactions between solid fuel use and age, which indicated that stronger association between solid fuel use for cooking and stroke in participants aged over 60 years old. We did not find a significant association between solid fuel use for heating and stroke, possibly because heating is only used during the cold season, and ventilation in heating is more common than in cooking (20). Previous studies on household air pollution have predominantly focused on women (26–28); hence, they might have considered women to be exposed to more household air pollution than men. However, the subgroup analysis in this study indicated that the risk of household solid fuel use for cooking on stroke was higher in men than in women, possibly because men in rural areas frequently burn coal or wood during the time that women cook meals. The exposure contents and levels of household air pollution in men may not be lower than those in women. Consistent with previous studies (12, 20), we also found solid cooking fuel to be associated with an increased risk of stroke in smokers than in non-smokers. We predicted that other types of interaction may exist between smoking status and solid fuel use.

Compared with previous studies, the strengths of this study include the following: it is the first comprehensive cohort study to include both male and female smokers and non-smokers, covering 450 urban and rural areas across China. The cause-result relationship between household solid fuel use and incident stroke was reliable in this study for a comparatively long follow-up period. The use of household solid fuel for different purposes demonstrated varying associations with stroke. Alterations in fuel use for cooking during the follow-up period also changed the effect on stroke incidence. Notwithstanding, this study has certain limitations. First, we used self-reported data on non-fatal stroke diagnosed by a doctor as the study outcome, there was no verification that these were real stroke cases. Although the follow-ups in this study were all door-to-door interviews by first positioning a participant's home address in baseline, thus the self-reported disease were reliable with limited missing data, further epidemiological studies should make more precise classifications of the outcome. Second, the diagnosis of the cases relied on self-reporting and only reported during periodical follow-up survey, the cases occurrence information was not real time information. Third, we only used two categories of fuel-use types for household air-pollution exposure instead of measuring personal exposure levels owing to the large population size. Household solid fuel use may not be popular among city populations. Forth, original follow-up data in 2015 for stroke incident only included 430 incident participants, for the rest participants, we categorized them as no incident stroke because we cannot distinguish if they had no incident stroke or were failed to follow-up; which might affect the results calculated in this study. Fifth, the composition of household solid fuel use was not considered because of the lack of related data and materials, and further studies to explore different sources and compositions of solid fuel use and stroke should be conducted to provide more precise explanations as well as biological mechanisms.

## Conclusion

The use of household solid fuel for cooking is positively associated with the incidence of stroke. Effective measures to improve the household cooking environment may be necessary to prevent stroke incidence.

## Data availability statement

The datasets presented in this study can be found in online repositories. The names of the repository/repositories and accession number(s) can be found at: http://charls.pku.edu.cn/index.htm.

## Ethics statement

The studies involving human participants were reviewed and approved by Ethics Committee of Peking University. The patients/participants provided their written informed consent to participate in this study.

## Author contributions

HZ, YX, and HS conducted the analysis process and wrote the paper. QC reviewed and revised the paper. YZ designed and revised the study. All authors contributed to the article and approved the submitted version.

## Funding

This work was supported by the National Natural Science Foundation of China (No. 82103791), the Natural Science Foundation of Liaoning Province (No. 2020MS172), and the 345 Talent Project of Shengjing Hospital of China Medical University (No. M0294).

## Conflict of interest

The authors declare that the research was conducted in the absence of any commercial or financial relationships that could be construed as a potential conflict of interest.

## Publisher's note

All claims expressed in this article are solely those of the authors and do not necessarily represent those of their affiliated organizations, or those of the publisher, the editors and the reviewers. Any product that may be evaluated in this article, or claim that may be made by its manufacturer, is not guaranteed or endorsed by the publisher.

## Abbreviations

PM_2.5_: particulate matter with a diameter <2.5 μm
HR: hazard ratio
CI: confidence interval
BMI: Body mass index.
